# Use of Split‐Intein Proteins to Design a Small Molecule Biosensor in Plants

**DOI:** 10.1111/pbi.70523

**Published:** 2025-12-31

**Authors:** Brandon A. Boone, Bal Maharjan, Van C. Nguyen, Jerry M. Parks, Tomás A. Rush, Carrie A. Eckert, Jin‐Gui Chen, Paul E. Abraham, Xiaohan Yang

**Affiliations:** ^1^ Biosciences Division Oak Ridge National Laboratory Oak Ridge Tennessee USA; ^2^ Department of Chemistry and Physics Western Carolina University Cullowhee North Carolina USA

Understanding how plants perceive their environment is fundamental to advancing agricultural productivity and sustainability. Many biological small molecules, including those involved in microbial recognition, act rapidly at the plant cell surface, but the absence of tools to visualise these dynamics has limited our ability to dissect plant–microbe communication. To address this gap, we sought to create a genetically encoded biosensor that couples ligand‐induced protein dimerization with the production of a fluorescent reporter. Inteins are peptide regions that excise themselves from precursor proteins and ligate the flanking chains (exteins). When each half of a split intein is fused to one of two dimerizing proteins, ligand binding brings them into proximity, inducing intein splicing and ligation of flanking extein sequences (Kang et al. 2022). Similar to previous studies, we split the yeast vacuolar ATPase subunit 1 (VMA1) intein, creating a protein biosensor that produces eGFP upon protein dimerization after ligand binding (Figure 1A) (Mootz et al. 2003). Specifically, eGFP halves (i.e., non‐functional N‐ and C‐terminal GFP fragments) were fused to the intein halves, resulting in two fusion proteins: N‐terminal GFP::N‐terminal intein and C‐terminal intein::C‐terminal GFP (Figure 1A).

**FIGURE 1:**
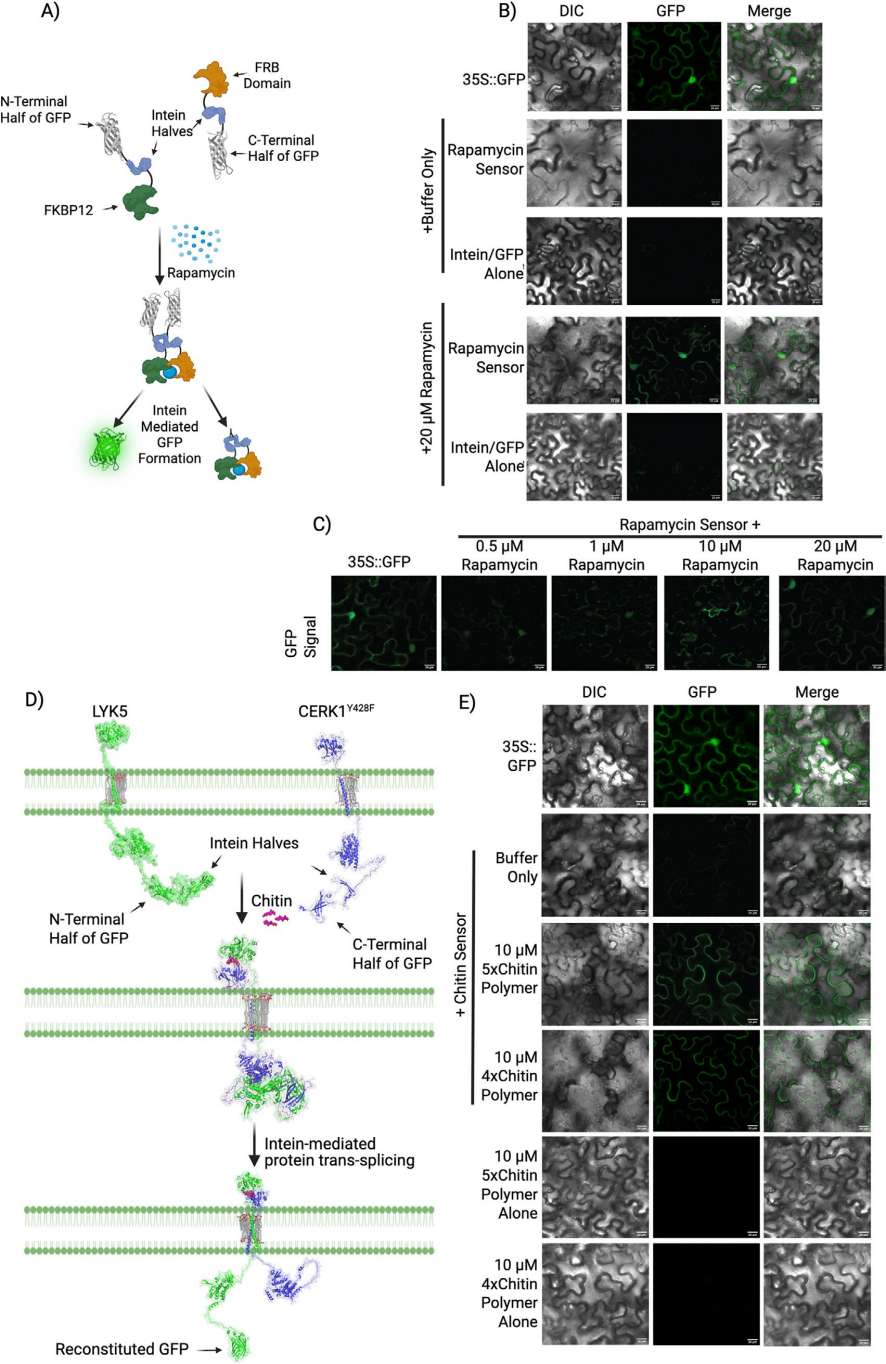
Split‐intein biosensor for detecting small molecules. (A) Model depicting FRB/FKBP12 with VMA1 split inteins (rapamycin sensor) for the creation of GFP. Made using BioRender.com. (B) GFP signal of GFP positive control and rapamycin sensor or split intein control in tobacco leaves. (C) GFP signal of rapamycin sensor with multiple concentrations of rapamycin. (D) AlphaFold3 models of the LYK5/CERK1^Y428F^ chitin sensor components before chitin binding, during chitin‐induced dimerization, and after intein‐mediated trans‐splicing. (E) GFP signal of tobacco leaves expressing chitin sensor alone or with chitin polymers, along with a GFP positive control. All scale bars in microscopy images are 20 μM.

As an initial demonstration, we designed a biosensor for the detection of rapamycin, which induces dimerization between FKBP12 protein and the FRB domain of the human mTOR complex (Saxton and Sabatini 2017). FKBP12 and the FRB domain were attached to the N‐ and C‐terminal intein/eGFP constructs, respectively, creating a biosensor that dimerizes in the presence of rapamycin, juxtaposing intein fragments and initiating trans‐splicing to produce eGFP (Figure 1A) (Saxton and Sabatini 2017). For experimental validation, sensor DNA constructs were transiently expressed in *Nicotiana benthamiana* leaves via *Agrobacterium*‐mediated infiltration. Fluorescence was observed in leaves that received rapamycin and the biosensor construct, at rapamycin concentrations as low as 500 nM and within 40 min of exposure, while no signal was detected in leaves expressing the biosensor alone, intein‐eGFP only, or buffer‐only controls (Figure 1B,C and Figure S1B). This result was validated across biological replicates as fluorescence was significantly increased compared to controls (Figure S1A). To verify that fluorescence output required intein splicing activity, we introduced point mutations (C284A and N737A) into the VMA1 intein sequence that abolish trans‐splicing activity (Anraku and Satow 2009). Expression of these inactive constructs failed to generate fluorescence in the presence of rapamycin (Figure S2A,B), demonstrating intein splicing activity is essential for signal generation. To rule out nonspecific reconstitution of eGFP fragments, we tested a bimolecular fluorescence complementation (BiFC) mimic in which FKBP12 and the FRB domain were directly fused to eGFP fragments without intein halves (Figure S2A,B). This construct did not produce fluorescence under the same conditions used for the rapamycin sensor (20 μM rapamycin), confirming fluorescence was not due to spontaneous eGFP fragment association.

Having validated our split‐intein biosensor using an orthogonal system, we applied this strategy to a biologically meaningful target: chitin. Chitin, an oligomer of β‐(1,4)‐linked N‐acetyl‐D‐glucosamine (GlcNAc) units, comprises the extracellular matrices of many fungi and insects and serves as conserved microbe‐associated molecular patterns (MAMPs) (Lv et al. 2023). Chitin perception is one of the earliest molecular events in plant innate responses to fungal pathogens, making it a valuable candidate for in vivo biosensing. A chitin biosensor could provide early detection of positive or negative interactions between plants and microbes, insects, or other chitin‐producing organisms. In *Arabidopsis thaliana* , the primary proteins that recognise chitin and activate downstream signalling pathways are LysM‐domain receptor‐like kinases LYK5 and CERK1 (Cao et al. 2014). CERK1 and LYK5 bind chitin at the plasma membrane using extracellular domains, inducing dimerization between CERK1 and LYK5 that activates immune signalling cascades (Yang et al. 2022).

To adapt our biosensor framework for chitin detection, we leveraged this chitin‐sensing pathway by attaching two biosensor halves (N‐terminal eGFP::N‐terminal intein and C‐terminal intein::C‐terminal eGFP) to LYK5 and CERK1, respectively, creating a chitin sensor (Figure 1D and Figure S3A). If chitin detection occurs, eGFP will reassemble directly attached to LYK5, which is anchored in the plant cell membrane (Figure 1D). To avoid potential immune signalling response in tobacco leaves, a mutant form of CERK1 (CERK1^Y428F^), which binds chitin and forms a heterodimeric complex with LYK5 but is unable to activate downstream signalling pathways, was used instead of wild‐type CERK1 (Liu et al. 2018). To assess potential folding issues and predict interactions of the intein/eGFP halves, we modelled the LYK5 and CERK1^Y428F^ intein::eGFP protein constructs separately, as pre‐ and post‐excision complexes, and without inteins (controls), using AlphaFold3 (Figure 1D and Figures S4–S9) (Abramson et al. 2024). These models provide evidence that chitin binding by LYK5 and CERK1^Y428F^ will induce interactions between the intein and eGFP halves, resulting in LYK5::eGFP protein production, and that the intein is not predicted to inhibit this process. Chitin sensor DNA constructs were transiently expressed in *Nicotiana benthamiana* using *Agrobacterium*‐mediated transformation. After 3 days, leaves were treated with purified chitin polymers or buffer‐only and analysed 1 h later using confocal microscopy. In contrast to buffer‐only controls, the chitin sensor generated fluorescence only in the presence of chitin tetramers, pentamers, or octamers and at concentrations as low as 1 μM and detected within 10 min of chitin application, consistent with in vivo chitin sensing (Figure 1E; Figures S10A,B and S11A,C) (Zhang et al. 2002). Fluorescence signal was significantly higher than controls when quantified across biological replicates and when compared across individual leaf images, reinforcing the reliability and specificity of the chitin sensor (Figures S10A and S11B). Notably, fluorescence was localised to cell periphery, consistent with the expected plasma membrane localization of LYK5 and CERK1^Y428F^ protein complexes (Figure 1E). Therefore, the chitin sensor consistently and rapidly generated chitin‐induced fluorescence localised as intended by the experimental design (Figure 1D). These results demonstrate a split‐intein biosensor capable of detecting biologically important receptor‐ligand interactions in plants. This generalizable biosensor could be applied to detect small‐molecule stimuli or to regulate cellular behaviour by producing numerous protein outputs, including transcription factors, gene‐editing enzymes, and signalling proteins. These possibilities provide numerous opportunities to utilise this technology across plant species and applications.

## Author Contributions

X.Y. supervised and edited the manuscript. B.A.B. designed experiments, analysed data, and wrote the manuscript. B.M., V.C.N., and J.M.P. performed experiments. B.M., C.A.E., P.E.A., and J.‐G.C. revised the manuscript. T.A.R. provided chitin polymers and experimental input. J.M.P. performed protein structural modeling and revised the manuscript.

## Funding

This work was supported by the U.S. Department of Energy, FWP ERKPA17.

## Conflicts of Interest

The authors declare no conflicts of interest.

## Supporting information


**Data S1:** pbi70523‐sup‐0001‐Supinfo1.zip.

**Data S2:** pbi70523‐sup‐0002‐Supinfo2.docx.

## Data Availability

The data supporting the findings of this study are available in the Supporting Information–S1 and S2 of this article.
