# Supplementary material for: Use of Split‐Intein Proteins to Design a Small Molecule Biosensor in Plants
Source: Plant Biotechnol J. 2025 Dec 31;24(4):2745–7. doi: 10.1111/pbi.70523 (PMC13140391; doi:10.1111/pbi.70523)
Supplement: Supplementary file 2 — Data S2: pbi70523‐sup‐0002‐Supinfo2.docx. [file PBI-24-2745-s002.docx]

**Plant Biotechnology Journal: Brief communication**

**Use of split-intein proteins to design a small molecule biosensor in plants**

Brandon A. Boone^1^, Bal Maharjan^1^, Van C. Nguyen^1^, Jerry M. Parks^1^, Tomás A. Rush^1^, Carrie A. Eckert^1^, Jin-Gui Chen^1^, Paul E. Abraham^1^, Xiaohan Yang^1,^*

^1^ Biosciences Division, Oak Ridge National Laboratory, Oak Ridge, TN, 37831, USA

*Correspondence: [yangx@ornl.gov](mailto:yangx@ornl.gov)

**Supplemental Materials and Methods**

**Plant materials, growth conditions, and transition plant expression**

Tobacco (*Nicotiana benthamiana*) plants were grown in greenhouse growth chambers at 16 hours of light and 8 hours of dark. Tobacco plants at ~3-4 weeks of age were used for transient expression experiments following previously described methods (Sparkes *et al.*, 2006). Infiltration buffer (10 mM MES pH 5.6, 10 mM MgCl_2_, and 100 μM acetosyringone) was used to dilute ~0.6 OD of *Agrobacterium* (GV3101) transformed with tDNA plasmids containing DNA constructs.

**Microscopy experiments and small-molecule addition to plants**

Microscopy analysis for all experiments was performed on the third day of the transient infiltration in tobacco leaves and after 1 hour of treatment with rapamycin, chitin, or buffer only with the exception of time course experiments. Microscopy experiments were performed on a Zeiss LSM 710 confocal microscope. All experiments were performed using 20X magnification settings. Addition of any small molecules were added 1 hour prior to microscopy analysis including buffer-only controls, rapamycin, and chitin polymers. Buffer controls were new infiltration buffers compared to rapamycin or chitin polymers diluted in the same infiltration buffer. Rapamycin was originally diluted in 100% DMSO, but stock solutions were created at ~1 mM concentration then diluted into infiltration buffer for experiments at the designated concentrations in figures. Chitin oligomers obtained from IsoSep (https://www.isosep.com/products/4512-0010.html and https://www.isosep.com/products/5514-0050.html) were diluted in ethanol at 100 μM for stock solutions and then diluted into infiltration buffer at concentrations listed in figures. All image analysis was performed using ImageJ software.

**Time course experiments**

Microscopy analysis for all-time-course experiments was performed on the third day after transient infiltration in tobacco leaves. Imaging was conducted using a Zeiss LSM 980 confocal microscope with a fixed 10X magnification. In the rapamycin experiment, a 20 µM solution of rapamycin was infiltrated into tobacco leaves, and samples were imaged at 0 min, 60 min, 120 min, and 24 hours post-infiltration. For the chitin experiment, a 10 µM solution of chitin was used, with imaging at 0 minutes, 10 minutes, 40 minutes, and 60 minutes post-infiltration.

**Graphs and Statistics**

All graphs are created using GraphPad Prism Software. All statistical analyses were also analyzed using GraphPad Prism software and are specifically mentioned in figure legends. For image analyses, image intensity measured from a 500x500 box was quantified across images from n=3 biological replicates (5 leaves per biological replicate and each replicate is from different days and using different plants and new agrobacteria) using ImageJ, image analysis software. This intensity was then plotted in box plots and compared statistically using One-way ANOVA with post hoc analysis. For the line plots of individual chitin sensor images, a line of the same length was drawn across the images (white line in the figure), and mean intensity values across that line were plotted in a line plot for each image using ImageJ software. No change in intensity was performed on the raw values.

**Construct design and cloning**

All cloning was performed using a modular cloning technique (Čermák *et al.*, 2017). Briefly, all constructs were cloned into either of four modules (pMODs A, B, C’, or D). These modules contain PaqCI sites that share homology to PaqCI sites in the tDNA plasmid (pTRANS210), which was used for transient expression experiments. The intein protein from yeast gene *VMA1* was split and attached to split eGFP halves in pMODA (N-terminal intein and N-terminal GFP) or pMODB (C-terminal intein and C-terminal GFP) containing BsmBI restriction enzyme sites to allow easy swapping of small ligand binding, protein domains (i.e., FRB and FKBP12). For the function of the intein construct, a point mutation was added to the eGFP coding sequence (I129C). For the mutant rapamycin sensor, new split intein/split GFP pMODs were ordered mimicking the original rapamycin sensor, split intein/split GFP pMODs except that they contain the two point mutations corresponding to the C284A and N737A mutations in the VMA1 intein coding sequence (Anraku and Satow, 2009). The BiFC mimic construct was also ordered in pMODA and pMODB for the N-terminal GFP and C-terminal GFP halves respectively but without the VMA1 intein and directly attached to the coding sequence of either the FRB domain or the FKBP12 protein.

For the chitin sensor, the construct design was changed wherein the N-terminal GFP half was attached directly to LYK5 followed by the VMA1 intein N-terminal half. CERK1^Y428F^ was linked to the C-terminal half of VMA1 intein followed by the C-terminal half of GFP similar to the rapamycin sensor.

**AlphaFold 3 modeling**

Protein structural models of the CERK1-based chitin sensor were generated with a modified version of AlphaFold 3 (Abramson, 2024) (https://github.com/Kuhlman-Lab/alphafold3) that uses ColabFold (Mirdita, 2022) and the MMseqs2 server (Steinegger, 2017) to generate multiple sequence alignments. Although AlphaFold 3 does not account for membrane environments, a small number of 1-palmitoyl-2-oleoyl-sn-glycero-3-phosphocholine (POPC) lipids (CCD code: POV) were included during modeling of the transmembrane domains of LYK5 and CERK1. In some cases, portions of the proteins occupied regions that would overlap with a realistic lipid bilayer, so folded domains were moved manually using PyMOL (The PyMOL Molecular Graphics System, Version 2.5.2 Schrödinger, LLC.) to place them in appropriate extracellular or intracellular orientations relative to the membrane. Flexible linkers were then removed from the models and reintroduced using PDBfixer version 1.11 (<https://github.com/openmm/pdbfixer>). We also generated a “control” model consisting of LYK5 and CERK1 fused directly to each eGFP half. Like the complete sensor, the control model maintains interactions between LYK5, chitin, and CERK1 on the extracellular side and between the two eGFP halves on the intracellular side (Figure S9).

To provide quantitative estimates of model accuracy, various confidence scores were analyzed for individual folded domains of the interacting components of the chitin sensor. Confidence scores include predicted local distance difference test (pLDDT), predicted template modeling score (pTM), interface predicted template modeling score (ipTM), chain_ipTM, and actual interface pTM (actifpTM) (Varga *et al.*, 2025). Briefly, pLDDT estimates per-atom local confidence, pTM estimates the accuracy of the global backbone topology, ipTM provides a confidence estimate of interchain contacts in protein-protein interactions, chain_ipTM estimates the accuracy of the interprotein interface for each chain pair, and actifpTM focuses on residues that participate in protein-protein interactions while omitting flanking regions. CIF files for all models are provided as Supporting Material.

**Protein sequences used in this study (N-terminus to C-terminus)**

FKBP12: MGVQVETISPGDGRTFPKRGQTCVVHYTGMLEDGKKFDSSRDRNKPFKFMLGKQEVIRGWEEGVAQMSVGQRAKLTISPDYAYGATGHPGIIPPHATLVFDVELLKLE

FRB Domain:

MRVAILWHEMWHEGLEEASRLYFGERNVKGMFEVLEPLHAMMERGPQTLKETSFNQAYGRDLMEAQEWCRKYMKSGNVKDLTQAWDLYYHVFRRISYPAFLYKVVDSRGGGGSGGGGS

VMA1 Intein N-terminus:

CFAKGTNVLMADGSIECIENIEVGNKVMGKDGRPREVIKLPRGRETMYSVVQKSQHRAHKSDSSREVPELLKFTCNATHELVVRTPRSVRRLSRTIKGVEYFEVITFEMGQKKAPDGRIVELVKEVSKSYPISEGPERANELVESYRKASNKAYFEWTIEARDLSLLGSHVRKATYQTYAPILY

VMA1 Intein C-terminus:

DVLLNVLSKCAGSKKFRPAPAAAFARECRGFYFELQELKEDDYYGITLSDDSDHQFLLANQVVVHN

eGFP:

MVSKGEELFTGVVPILVELDGDVNGHKFSVSGEGEGDATYGKLTLKFICTTGKLPVPWPTLVTTLTYGVQCFSRYPDHMKQHDFFKSAMPEGYVQERTIFFKDDGNYKTRAEVKFEGDTLVNRIILKGCDFKEDGNILGHKLEYNYNSHNVYIMADKQKNGIKVNFKIRHNIEDGSVQLADHYQQNTPIGDGPVLLPDNHYLSTQSALSKDPNEKRDHMVLLEFVTAAGITLGMDELYK

LYK5:

MAACTLHALSVTLFLLLFFAVSPAKAQQPYVNNHQLACEVRVYDNITNGFTCNGPPSCRSYLTFWSQPPYNTADSIAKLLNVSAAEIQSINNLPTATTRIPTRELVVIPANCSCSSSSGGFYQHNATYNLSGNRGDETYFSVANDTYQALSTCQAMMSQNRYGERQLTPGLNLLVPLRCACPTAKQTTAGFKYLLTYLVAMGDSISGIAEMFNSTSAAITEGNELTSDNIFFFTPVLVPLTTEPTKIVISPSPPPPPVVATPPQTPVDPPGSSSSHKWIYIGIGIGAGLLLLLSILALCFYKRRSKKKSLPSSLPEENKLFDSSTKQSIPTTTTTQWSIDLSNSSEAFGLKSAIESLTLYRFNDLQSATSNFSDENRIKGSVYRATINGDDAAVKVIKGDVSSSEINLLKKLNHSNIIRLSGFCIREGTSYLVFEYSENGSISDWLHSSGKKSLTWKQRVEIARDVAEALDYLHNYITPPHIHKNLESTNILLDSNFRAKIANFGVARILDEGDLDLQLTRHVEGTQGYLAPEYVENGVITSKLDVFAFGVAVLELLSGREAVTIHKKKEGEEEVEMLCKVINSVLGGENVREKLKEFMDPSLGNEYPLELAYTMAQLAKSCVATDLNSRPSVTQVLTTLSMIVSSSIDWEPSDDLLRSGSLGN

CERK1^Y428F^:

MKLKISLIAPILLLFSFFFAVESKCRTSCPLALASYYLENGTTLSVINQNLNSSIAPYDQINFDPILRYNSNIKDKDRIQMGSRVLVPFPCECQPGDFLGHNFSYSVRQEDTYERVAISNYANLTTMESLQARNPFPATNIPLSATLNVLVNCSCGDESVSKDFGLFVTYPLRPEDSLSSIARSSGVSADILQRYNPGVNFNSGNGIVYVPGRDPNGAFPPFKSSKQDGVGAGVIAGIVIGVIVALLLILFIVYYAYRKNKSKGDSFSSSIPLSTKADHASSTSLQSGGLGGAGVSPGIAAISVDKSVEFSLEELAKATDNFNLSFKIGQGGFGAVYYAELRGEKAAIKKMDMEASKQFLAELKVLTRVHHVNLVRLIGYCVEGSLFLVYEYVENGNLGQHLHGSGREPLPWTKRVQIALDSARGLEFIHEHTVPVYVHRDIKSANILIDQKFRAKVADFGLTKLTEVGGSATRGAMGTFGYMAPETVYGEVSAKVDVYAFGVVLYELISAKGAVVKMTEAVGEFRGLVGVFEESFKETDKEEALRKIIDPRLGDSYPFDSVYKMAELGKACTQENAQLRPSMRYIVVALSTLFSSTGNWDVGNFQNEDLVSLMSGR

LYK5-Flag Tag-N Terminal GFP-N-terminal Intein:

MAACTLHALSVTLFLLLFFAVSPAKAQQPYVNNHQLACEVRVYDNITNGFTCNGPPSCRSYLTFWSQPPYNTADSIAKLLNVSAAEIQSINNLPTATTRIPTRELVVIPANCSCSSSSGGFYQHNATYNLSGNRGDETYFSVANDTYQALSTCQAMMSQNRYGERQLTPGLNLLVPLRCACPTAKQTTAGFKYLLTYLVAMGDSISGIAEMFNSTSAAITEGNELTSDNIFFFTPVLVPLTTEPTKIVISPSPPPPPVVATPPQTPVDPPGSSSSHKWIYIGIGIGAGLLLLLSILALCFYKRRSKKKSLPSSLPEENKLFDSSTKQSIPTTTTTQWSIDLSNSSEAFGLKSAIESLTLYRFNDLQSATSNFSDENRIKGSVYRATINGDDAAVKVIKGDVSSSEINLLKKLNHSNIIRLSGFCIREGTSYLVFEYSENGSISDWLHSSGKKSLTWKQRVEIARDVAEALDYLHNYITPPHIHKNLESTNILLDSNFRAKIANFGVARILDEGDLDLQLTRHVEGTQGYLAPEYVENGVITSKLDVFAFGVAVLELLSGREAVTIHKKKEGEEEVEMLCKVINSVLGGENVREKLKEFMDPSLGNEYPLELAYTMAQLAKSCVATDLNSRPSVTQVLTTLSMIVSSSIDWEPSDDLLRSGSLGNDYKDDDDKDYKDDDDKDYKDDDDKASNNGNGRNGMV

SKGEELFTGVVPILVELDGDVNGHKFSVSGEGEGDATYGKLTLKFICTTGKLPVPWPTLVTTLTYGVQCFSRYPDHMKQHDFFKSAMPEGYVQERTIFFKDDGNYKTRAEVKFEGDTLVNRIILKGCFAKGTNVLMADGSIECIENIEVGNKVMGKDGRPREVIKLPRGRETMYSVVQKSQHRAHKSDSSREVPELLKFTCNATHELVVRTPRSVRRLSRTIKGVEYFEVITFEMGQKKAPDGRIVELVKEVSKSYPISEGPERANELVESYRKASNKAYFEWTIEARDLSLLGSHVRKATYQTYAPILY

CERK1^Y428F^-Flag Tag-C terminal Intein-C terminal GFP:

MKLKISLIAPILLLFSFFFAVESKCRTSCPLALASYYLENGTTLSVINQNLNSSIAPYDQINFDPILRYNSNIKDKDRIQMGSRVLVPFPCECQPGDFLGHNFSYSVRQEDTYERVAISNYANLTTMESLQARNPFPATNIPLSATLNVLVNCSCGDESVSKDFGLFVTYPLRPEDSLSSIARSSGVSADILQRYNPGVNFNSGNGIVYVPGRDPNGAFPPFKSSKQDGVGAGVIAGIVIGVIVALLLILFIVYYAYRKNKSKGDSFSSSIPLSTKADHASSTSLQSGGLGGAGVSPGIAAISVDKSVEFSLEELAKATDNFNLSFKIGQGGFGAVYYAELRGEKAAIKKMDMEASKQFLAELKVLTRVHHVNLVRLIGYCVEGSLFLVYEYVENGNLGQHLHGSGREPLPWTKRVQIALDSARGLEFIHEHTVPVYVHRDIKSANILIDQKFRAKVADFGLTKLTEVGGSATRGAMGTFGYMAPETVYGEVSAKVDVYAFGVVLYELISAKGAVVKMTEAVGEFRGLVGVFEESFKETDKEEALRKIIDPRLGDSYPFDSVYKMAELGKACTQENAQLRPSMRYIVVALSTLFSSTGNWDVGNFQNEDLVSLMSGRDYKDDDDKDYKDDDDKDYKDDDDKGNNGGNNDVDVLLNVLSKCAGSKKFRPAPAAAFARECRGFYFELQELKEDDYYGITLSDDSDHQFLLANQVVVHNCDFKEDGNILGHKLEYNYNSHNVYIMADKQKNGIKVNFKIRHNIEDGSVQLADHYQQNTPIGDGPVLLPDNHYLSTQSALSKDPNEKRDHMVLLEFVTAAGITLGMDELYK

LYK5-Flag Tag-full GFP:

MAACTLHALSVTLFLLLFFAVSPAKAQQPYVNNHQLACEVRVYDNITNGFTCNGPPSCRSYLTFWSQPPYNTADSIAKLLNVSAAEIQSINNLPTATTRIPTRELVVIPANCSCSSSSGGFYQHNATYNLSGNRGDETYFSVANDTYQALSTCQAMMSQNRYGERQLTPGLNLLVPLRCACPTAKQTTAGFKYLLTYLVAMGDSISGIAEMFNSTSAAITEGNELTSDNIFFFTPVLVPLTTEPTKIVISPSPPPPPVVATPPQTPVDPPGSSSSHKWIYIGIGIGAGLLLLLSILALCFYKRRSKKKSLPSSLPEENKLFDSSTKQSIPTTTTTQWSIDLSNSSEAFGLKSAIESLTLYRFNDLQSATSNFSDENRIKGSVYRATINGDDAAVKVIKGDVSSSEINLLKKLNHSNIIRLSGFCIREGTSYLVFEYSENGSISDWLHSSGKKSLTWKQRVEIARDVAEALDYLHNYITPPHIHKNLESTNILLDSNFRAKIANFGVARILDEGDLDLQLTRHVEGTQGYLAPEYVENGVITSKLDVFAFGVAVLELLSGREAVTIHKKKEGEEEVEMLCKVINSVLGGENVREKLKEFMDPSLGNEYPLELAYTMAQLAKSCVATDLNSRPSVTQVLTTLSMIVSSSIDWEPSDDLLRSGSLGNDYKDDDDKDYKDDDDKDYKDDDDKASNNGNGRNGMV

SKGEELFTGVVPILVELDGDVNGHKFSVSGEGEGDATYGKLTLKFICTTGKLPVPWPTLVTTLTYGVQCFSRYPDHMKQHDFFKSAMPEGYVQERTIFFKDDGNYKTRAEVKFEGDTLVNRIILKGCDFKEDGNILGHKLEYNYNSHNVYIMADKQKNGIKVNFKIRHNIEDGSVQLADHYQQNTPIGDGPVLLPDNHYLSTQSALSKDPNEKRDHMVLLEFVTAAGITLGMDELYK

CERK1^Y428F^-Flag Tag-C terminal Intein:

MKLKISLIAPILLLFSFFFAVESKCRTSCPLALASYYLENGTTLSVINQNLNSSIAPYDQINFDPILRYNSNIKDKDRIQMGSRVLVPFPCECQPGDFLGHNFSYSVRQEDTYERVAISNYANLTTMESLQARNPFPATNIPLSATLNVLVNCSCGDESVSKDFGLFVTYPLRPEDSLSSIARSSGVSADILQRYNPGVNFNSGNGIVYVPGRDPNGAFPPFKSSKQDGVGAGVIAGIVIGVIVALLLILFIVYYAYRKNKSKGDSFSSSIPLSTKADHASSTSLQSGGLGGAGVSPGIAAISVDKSVEFSLEELAKATDNFNLSFKIGQGGFGAVYYAELRGEKAAIKK

MDMEASKQFLAELKVLTRVHHVNLVRLIGYCVEGSLFLVYEYVENGNLGQHLHGSGREPLPWTKRVQIALDSARGLEFIHEHTVPVYVHRDIKSANILIDQKFRAKVADFGLTKLTEVGGSATRGAMGTFGYMAPETVYGEVSAKVDVYAFGVVLYELISAKGAVVKMTEAVGEFRGLVGVFEESFKETDKEEALRKIIDPRLGDSYPFDSVYKMAELGKACTQENAQLRPSMRYIVVALSTLFSSTGNWDVGNFQNEDLVSLMSGRDYKDDDDKDYKDDDDKDYKDDDDKGNNGGNNDVDVLLNVLSKCAGSKKFRPAPAAAFARECRGFYFELQELKEDDYYGITLSD

DSDHQFLLANQVVVHN

**DNA sequences used in this study**

pMODA containing N-terminal GFP/N-terminal intein with BsmBI sites for protein:

GCCTTGACGGCAAAATCCCTTAACGTGAGTTTTCGTTCCACTGAGCGTCAGACCCCGTAGAAAAGATCAAAGGATCTTCTTGAGATCCTTTTTTTCTGCGCGTAATCTGCTGCTTGCAAACAAAAAAACCACCGCTACCAGCGGTGGTTTGTTTGCCGGATCAAGAGCTACTAACTCTTTTTCCGAAGGTAACTGGCTTCAGCAGAGCGCAGATACCAAATACTGTTCTTCTAGTGTAGCCGTAGTTAGGCCACCACTTCAAGAACTCTGTAGCACCGCCTACATACCTCGCTCTGCTAATCCTGTTACCAGTGGCTGCTGCCAGTGGCGATAAGTCGTGTCTTACCGGGTTGGACTCAAGACGATAGTTACCGGATAAGGCGCAGCGGTCGGGCTGAACGGGGGGTTCGTGCTCACAGCCCAGCTTGGAGCGAACGACCTACACCGAACTGAGATACCTACAGCGTGAGCTATGAGAAAGCGCCACGCTTCCCGAAGGGAGAAAGGCGGACAGGTATCCGGTAAGCGGCAGGGTCGGAACAGGAGAGCGCACGAGGGAGCTTCCAGGGGGAAACGCCTGGTATCTTTATAGTCCTGTCGGGTTTCGCCACCTCTGACTTGAGCGTCGATTTTTGTGATGCTCGTCAGGGGGGCGGAGCCTATGGAAAAACGCCAGCAACGCGGCCTTTTTACGGTTCCTGGCCTTTTGCTGGCCTTTTGCTCACATGTTCTTTCCTGCGTTATCCCCTGATTCTGTGGATAACCGTATTACCGCCTTTGAGTGAGCTGATACCGCTCGCCGCAGCCGAACGACCGAGCGCAGCGAGTCAGTGAGCGAGGAAGCGGAAGAGCGCCCACCTGCTCATGATCGGCTGAGACTTTTCAACAAAGGGTAATATCCGGAAACCTCCTCGGATTCCATTGCCCAGCTATCTGTCACTTTATTGTGAAGATAGTGGAAAAGGAAGGTGGCTCCTACAAATGCCATCATTGCGATAAAGGAAAGGCCATCGTTGAAGATGCCTCTGCCGACAGTGGTCCCAAAGATGGACCCCCACCCACGAGGAGCATCGTGGAAAAAGAAGACGTTCCAACCACGTCTTCAAAGCAAGTGGATTGATGTGATATCTCCACTGACGTAAGGGATGACGCACAATCCCACTATCCTTCGCAAGACCCTTCCTCTATATAAGGAAGTTCATTTCATTTGGAGAGAACACGGGGGACTCAAACATTACTCATTCACAAAACCATCTTAAAGCAACTACACAAGTCTTGAAATTTTCTCATATTTTCTATTTACTATATAAACTTTTAATCAAATCAAGATTAAAGTTAATTAAATGGTTTCAAAAGGTGAGGAACTTTTCACCGGCGTGGTACCGATTTTGGTCGAATTAGATGGCGACGTAAACGGTCACAAATTCAGTGTCTCCGGGGAGGGTGAGGGTGATGCAACCTACGGCAAGCTTACGTTAAAATTCATCTGCACAACCGGTAAGCTACCGGTCCCTTGGCCTACACTGGTGACAACACTGACATACGGGGTACAGTGCTTTAGTCGTTATCCAGATCATATGAAACAACATGATTTTTTTAAGAGTGCGATGCCAGAAGGGTATGTTCAAGAACGAACGATATTTTTCAAGGACGATGGGAATTACAAGACTAGAGCAGAGGTAAAGTTTGAGGGTGATACTCTCGTCAACCGAATCATCTTGAAGGGTTGCTTTGCAAAAGGTACCAATGTTTTGATGGCCGATGGGTCTATCGAATGTATTGAGAACATAGAAGTAGGGAACAAGGTCATGGGAAAGGACGGTCGACCGCGAGAAGTAATAAAATTGCCTAGAGGGAGGGAGACTATGTACTCAGTGGTGCAAAAAAGTCAACATAGGGCCCACAAATCTGATAGCAGCAGGGAAGTTCCTGAATTATTAAAGTTCACATGTAACGCCACCCATGAGCTAGTAGTCCGTACGCCGCGATCAGTGCGTCGACTTAGCCGAACTATTAAGGGCGTTGAATACTTCGAGGTCATAACTTTTGAAATGGGTCAGAAAAAAGCTCCTGATGGGAGAATTGTCGAGCTGGTAAAGGAGGTCAGTAAATCATATCCGATAAGTGAAGGCCCCGAACGAGCGAACGAACTAGTTGAGAGCTATCGTAAAGCGTCTAACAAAGCCTACTTTGAATGGACTATAGAAGCCCGTGACCTATCCCTGTTGGGTTCTCATGTCCGTAAGGCAACATACCAGACGTACGCCCCTATACTGTATGCGTCCAATAACGGGAACGGCAGGAACGGGTGAGACGTAGCGTCTCAATATGAAGATGAAGATGAAATATTTGGTGTGTCAAATAAAAAGCTTGTGTGCTTAAGTTTGTGTTTTTTTCTTGGCTTGTTGTGTTATGAATTTGTGGCTTTTTCTAATATTAAATGAATGTAAGATCTCATTATAATGAATAAACAAATGTTTCTATAATCCATTGTGAATGTTTTGTTGGATCTCTTCTGCAGCATATAACTACTGTATGTGCTATGGTATGGACTATGGAATATGATTAAAGATAAGATGGGCTCATAGAGTAAAACGAGGCGAGGGACCTATAAACCTCCCTTCATCATGCTATTTCATGATCTATTTTATAAAATAAAGATGTAGAAAAAAGTAAGCGTAATAACCGCAAAACAAATGATTTAAAACATGGCACATAATGAGGAGATTAAGTTCGGTTTACGTTTATTTTAGTACTAATTGTAACGTGAGACTACGTATCGGGAATCGCCTAATTAAAGCATTAATGCGAACCTGATTAGATTCACCGACCCTCCTATCGTGTCGACCTTTCTGTTTCTTAGAATTTTTTGGTAGTCTATGTACTAATAATGTCAGCTTCGTATTTATTTCATAAGCAATTTGCATTTGCAATTTGTTTTTTACTTTTATTTTTATTGTATTGTGGAATGTGGACTCGTACCAACATGAAGTTATATACCACCAAAAAAATTACAGTTAGTCAAAAGATTCACGAGTGAGAGCTACTTATGATTGTCTTTTACGTATATGTCTAATTGTCTATTTGCTCAATAATCTTTGTACTTTCTTTTGTCGTTGATAAAATCACAAAGTTCCAAAAGTAATCGAATGATTTGCTTTTAAGAAAAGAAGAGCTCAATAATTCAACATATATCTGTACACAGACGGACGTGGGCAGGTGATGAAAAGCCTTAGGGTCAGGTGGCACTTTTCGGGGAAATGTGCGATGAACCCCTATTTGTTTATTTTTCTAAATACATTCAAATATGTATCCGCTAATGAGACAATAACCCTGATAAATGCTTCAATTATATTGAAAAAGGAAGAGTATGAGTATCCAACATTTCCGTGTCGCCCTTATTCCCTTTTTTGCGGCCTTCTGCCTTCCTGTTTTTGCTCACCCAGAAACGCTGGTGAAAGTAAAAGATGCTGAAGATCAGTTGGGTGCCCGAGTGGGTTACATTGAACTGGATCTCAACAGCGGCAAAATCTTAGAGAGTTTTCGCCCCGAAGAACGCTTCCCAATGATGAGCACTTTCAAAGTTCTGCTATGTGGCGCGGTGTTATCCCGTATTGATGCTGGGCAAGAGCAACTCGGTCGCCGCATACACTATTCTCAGAATGACTTGGTTGAGTATTCACCAGTCACAGAAAAGCATCTTACGGATGGCATGACAGTAAGAGAATTATGTAGTGCTGCCATAACCATGAGTGATAACACTGCGGCCAACTTACTTCTGACAACGATTGGAGGACCGAAGGAGCTAACCGCTTTTTTGCACAACATGGGGGATCATGTAACTCGCCTTGATCGTTGGGAACCGGAGCTGAATGAAGCCATACCAAACGACGAGCGTGACACCACGATGCCTGTAGCAATGGCAACAACTCTCCGCAAACTATTAACTGGCGAACTACTTACTCTTGCTTCACGCCAGCAACTCATTGACTGGATGGAGGCGGACAAAGTTGCAGGACCACTTCTGCGCTCGGCACTTCCGGCTGGCTGGTTTATTGCTGATAAATCTGGAGCCGGTGAGCGTGGGTCTCGCGGTATCATTGCAGCACTGGGGCCAGATGGTAAGCCCTCCCGTATCGTAGTTATCTACACGACGGGGAGTCAGGCAACTATGGATGAACGAAATAGACAGATCGCTGAGATAGGTGCCTCACTGATTAAGCATTGGTAACTGTCAGACGAAGGGAAATAAATAGTAGCCCGCCTGATGTGCGGGCTTTTTTTTGGTGGTGGTTACCAGTGTCGCTTAGCTACCGTAGGTCCTCTTAGGCGGTCCGATTAGCGACATAGTCATCAAT

pMODB containing C-terminal intein/C-terminal GFP with BsmBI sites for protein:

ACCGAAGGAGCTAACCGCTTTTTTGCACAACATGGGGGATCATGTAACTCGCCTTGATCGTTGGGAACCGGAGCTGAATGAAGCCATACCAAACGACGAGCGTGACACCACGATGCCTGTAGCAATGGCAACAACTCTCCGCAAACTATTAACTGGCGAACTACTTACTCTTGCTTCACGCCAGCAACTCATTGACTGGATGGAGGCGGACAAAGTTGCAGGACCACTTCTGCGCTCGGCACTTCCGGCTGGCTGGTTTATTGCTGATAAATCTGGAGCCGGTGAGCGTGGCTCTCGCGGTATCATTGCAGCACTGGGGCCAGATGGTAAGCCCTCCCGTATCGTAGTTATCTACACGACGGGGAGTCAGGCAACTATGGATGAACGAAATAGACAGATCGCTGAGATAGGTGCCTCACTGATTAAGCATTGGTAACTGTCAGACGAAGGGAAATAAATAGTAGCCCGCCTGATGTGCGGGCTTTTTTTTGGTGGTGGTTACCAGTGTCGCTTAGCTACCGTAGGTCCTCTTAGGCGGTCCGATTAGCGACATAGTCATCAATGCCTTGACGGCAAAATCCCTTAACGTGAGTTTTCGTTCCACTGAGCGTCAGACCCCGTAGAAAAGATCAAAGGATCTTCTTGAGATCCTTTTTTTCTGCGCGTAATCTGCTGCTTGCAAACAAAAAAACCACCGCTACCAGCGGTGGTTTGTTTGCCGGATCAAGAGCTACTAACTCTTTTTCCGAAGGTAACTGGCTTCAGCAGAGCGCAGATACCAAATACTGTTCTTCTAGTGTAGCCGTAGTTAGGCCACCACTTCAAGAACTCTGTAGCACCGCCTACATACCTCGCTCTGCTAATCCTGTTACCAGTGGCTGCTGCCAGTGGCGATAAGTCGTGTCTTACCGGGTTGGACTCAAGACGATAGTTACCGGATAAGGCGCAGCGGTCGGGCTGAACGGGGGGTTCGTGCTCACAGCCCAGCTTGGAGCGAACGACCTACACCGAACTGAGATACCTACAGCGTGAGCTATGAGAAAGCGCCACGCTTCCCGAAGGGAGAAAGGCGGACAGGTATCCGGTAAGCGGCAGGGTCGGAACAGGAGAGCGCACGAGGGAGCTTCCAGGGGGAAACGCCTGGTATCTTTATAGTCCTGTCGGGTTTCGCCACCTCTGACTTGAGCGTCGATTTTTGTGATGCTCGTCAGGGGGGCGGAGCCTATGGAAAAACGCCAGCAACGCGGCCTTTTTACGGTTCCTGGCCTTTTGCTGGCCTTTTGCTCACATGTTCTTTCCTGCGTTATCCCCTGATTCTGTGGATAACCGTATTACCGCCTTTGAGTGAGCTGATACCGCTCGCCGCAGCCGAACGACCGAGCGCAGCGAGTCAGTGAGCGAGGAAGCGGGAGAGCGCCCACCTGCCAGGGGACTGAGACTTTTCAACAAAGGGTAATATCCGGAAACCTCCTCGGATTCCATTGCCCAGCTATCTGTCACTTTATTGTGAAGATAGTGGAAAAGGAAGGTGGCTCCTACAAATGCCATCATTGCGATAAAGGAAAGGCCATCGTTGAAGATGCCTCTGCCGACAGTGGTCCCAAAGATGGACCCCCACCCACGAGGAGCATCGTGGAAAAAGAAGACGTTCCAACCACGTCTTCAAAGCAAGTGGATTGATGTGATATCTCCACTGACGTAAGGGATGACGCACAATCCCACTATCCTTCGCAAGACCCTTCCTCTATATAAGGAAGTTCATTTCATTTGGAGAGAACACGGGGGACTCTAGACAAACATTACTCATTCACAAAACCATCTTAAAGCAACTACACAAGTCTTGAAATTTTCTCATATTTTCTATTTACTATATAAACTTTTAATCAAATCAAGATTAAAGTTAATTAATGAGACGATGCGTCTCCGGTAACAACGGCGGGAACAATGACGTGGACGTTCTTCTGAATGTGTTAAGTAAGTGCGCGGGCAGCAAAAAGTTCCGACCTGCTCCCGCCGCGGCCTTTGCAAGAGAATGCAGGGGGTTTTACTTCGAGCTTCAAGAGCTTAAGGAAGACGACTATTATGGCATCACCCTTTCTGACGACAGTGATCACCAGTTTCTCCTGGCCAATCAAGTAGTAGTCCACAACTGTGATTTCAAGGAAGACGGGAACATTCTGGGTCATAAGCTAGAGTATAACTACAACTCCCACAACGTTTACATAATGGCCGATAAGCAGAAAAACGGGATAAAAGTCAATTTTAAAATACGACATAACATAGAGGATGGGTCCGTCCAATTGGCAGACCACTATCAACAAAACACTCCGATTGGTGACGGTCCAGTCTTATTGCCTGATAACCATTACCTGAGCACTCAGTCAGCTCTAAGCAAAGACCCTAACGAAAAACGAGACCATATGGTGCTGCTGGAGTTCGTCACAGCAGCAGGGATCACCTTAGGAATGGATGAACTTTATAAGTAGATATGAAGATGAAGATGAAATATTTGGTGTGTCAAATAAAAAGCTTGTGTGCTTAAGTTTGTGTTTTTTTCTTGGCTTGTTGTGTTATGAATTTGTGGCTTTTTCTAATATTAAATGAATGTAAGATCTCATTATAATGAATAAACAAATGTTTCTATAATCCATTGTGAATGTTTTGTTGGATCTCTTCTGCAGCATATAACTACTGTATGTGCTATGGTATGGACTATGGAATATGATTAAAGATAAGATGGGCTCATAGAGTAAAACGAGGCGAGGGACCTATAAACCTCCCTTCATCATGCTATTTCATGATCTATTTTATAAAATAAAGATGTAGAAAAAAGTAAGCGTAATAACCGCAAAACAAATGATTTAAAACATGGCACATAATGAGGAGATTAAGTTCGGTTTACGTTTATTTTAGTACTAATTGTAACGTGAGACTACGTATCGGGAATCGCCTAATTAAAGCATTAATGCGAACCTGATTAGATTCACCGACCCTCCTATCGTGTCGACCTTTCTGTTTCTTAGAATTTTTTGGTAGTCTATGTACTAATAATGTCAGCTTCGTATTTATTTCATAAGCAATTTGCATTTGCAATTTGTTTTTTACTTTTATTTTTATTGTATTGTGGAATGTGGACTCGTACCAACATGAAGTTATATACCACCAAAAAAATTACAGTTAGTCAAAAGATTCACGAGTGAGAGCTACTTATGATTGTCTTTTACGTATATGTCTAATTGTCTATTTGCTCAATAATCTTTGTACTTTCTTTTGTCGTTGATAAAATCACAAAGTTCCAAAAGTAATCGAATGATTTGCTTTTAAGAAAAGAAGAGCTCAATAATTCAACATATATCTGTACACACTATGGAGGCTCCCGGTGACGCAGGTGATGAAAAGCCTTAGGGTCAGGTGGCACTTTTCGGGGAAATGTGCGATGAACCCCTATTTGTTTATTTTTCTAAATACATTCAAATATGTATCCGCTAATGAGACAATAACCCTGATAAATGCTTCAATTATATTGAAAAAGGAAGAGTATGAGTATCCAACATTTCCGTGTCGCCCTTATTCCCTTTTTTGCGGCCTTCTGCCTTCCTGTTTTTGCTCACCCAGAAACGCTGGTGAAAGTAAAAGATGCTGAAGATCAGTTGGGTGCCCGAGTGGGTTACATTGAACTGGATCTCAACAGCGGCAAAATCTTAGAGAGTTTTCGCCCCGAAGAACGCTTCCCAATGATGAGCACTTTCAAAGTTCTGCTATGTGGCGCGGTGTTATCCCGTATTGATGCTGGGCAAGAGCAACTCGGTCGCCGCATACACTATTCTCAGAATGACTTGGTTGAGTATTCACCAGTCACAGAAAAGCATCTTACGGATGGCATGACAGTAAGAGAATTATGTAGTGCTGCCATAACCATGAGTGATAACACTGCGGCCAACTTACTTCTGACAACGATTGGAGG

Gene block for LYK5 directly attached to N-terminal GFP and N-terminal intein for pMODA:

CACCTGCTCATGATCGGCTGAGACTTTTCAACAAAGGGTAATATCCGGAAACCTCCTCGGATTCCATTGCCCAGCTATCTGTCACTTTATTGTGAAGATAGTGGAAAAGGAAGGTGGCTCCTACAAATGCCATCATTGCGATAAAGGAAAGGCCATCGTTGAAGATGCCTCTGCCGACAGTGGTCCCAAAGATGGACCCCCACCCACGAGGAGCATCGTGGAAAAAGAAGACGTTCCAACCACGTCTTCAAAGCAAGTGGATTGATGTGATATCTCCACTGACGTAAGGGATGACGCACAATCCCACTATCCTTCGCAAGACCCTTCCTCTATATAAGGAAGTTCATTTCATTTGGAGAGAACACGGGGGACTCAAACATTACTCATTCACAAAACCATCTTAAAGCAACTACACAAGTCTTGAAATTTTCTCATATTTTCTATTTACTATATAAACTTTTAATCAAATCAAGATTAAAGTTAATTAAATGGCTGCGTGTACACTCCACGCGCTCTCAGTCACCTTATTCCTCCTTCTCTTCTTTGCCGTGTCACCGGCGAAAGCTCAGCAACCGTACGTCAACAACCACCAGCTCGCCTGCGAGGTCCGTGTCTACGACAACATAACCAACGGATTCACATGTAACGGCCCACCTTCTTGCCGCTCATACCTCACTTTCTGGTCTCAACCACCGTACAACACGGCGGACTCAATCGCCAAACTCCTCAACGTCTCCGCCGCAGAGATCCAATCAATCAACAACCTCCCCACAGCCACCACCAGAATCCCAACCCGTGAATTAGTCGTGATCCCAGCTAACTGCTCCTGCTCCTCCTCCAGCGGAGGATTTTACCAACACAACGCCACTTACAATCTCTCCGGTAACAGAGGAGATGAAACCTATTTCTCAGTGGCTAACGATACTTACCAAGCTTTATCCACGTGTCAAGCCATGATGTCACAAAACCGTTACGGCGAGAGACAACTAACCCCCGGCTTAAACCTCCTTGTTCCTCTCCGATGTGCTTGTCCCACCGCCAAACAAACCACCGCCGGATTTAAATATCTCCTGACTTACTTAGTCGCCATGGGAGATAGTATCTCCGGCATCGCCGAGATGTTCAACAGCACATCCGCCGCCATAACCGAAGGTAACGAGCTTACATCAGACAATATCTTCTTCTTCACACCGGTTCTAGTTCCTCTCACAACTGAACCTACCAAAATCGTTATATCTCCGTCGCCTCCTCCTCCACCCGTTGTTGCTACGCCGCCTCAAACGCCAGTTGATCCTCCGGGATCTTCTTCTTCTCACAAATGGATCTACATCGGAATTGGAATCGGAGCTGGTTTGCTTCTCTTACTCTCAATCTTAGCTCTCTGCTTCTACAAACGAAGGTCTAAGAAGAAGTCATTACCGTCGTCGTTGCCGGAGGAGAACAAGCTCTTTGATTCATCAACCAAACAATCTATTCCCACAACAACAACGACTCAATGGTCAATAGATTTATCCAATTCATCAGAAGCTTTCGGTTTAAAATCCGCCATAGAATCTCTAACACTATACAGATTCAACGATCTTCAATCAGCTACTTCGAATTTCAGCGACGAAAACAGAATCAAAGGCTCTGTTTATCGCGCGACAATCAACGGCGACGATGCCGCTGTGAAAGTGATCAAAGGAGATGTTTCTTCCTCTGAGATCAATCTTTTGAAGAAGCTAAATCATTCTAATATCATCCGTCTCTCAGGTTTCTGTATCCGTGAAGGAACATCGTACCTCGTCTTCGAGTATTCAGAGAATGGATCGATCAGTGATTGGCTTCACTCGTCGGGCAAGAAGAGTTTGACATGGAAACAGAGAGTTGAAATAGCAAGGGATGTGGCAGAGGCGTTAGATTATCTCCATAACTATATAACTCCACCTCATATTCATAAGAACTTGGAATCAACAAACATACTTCTGGATTCTAATTTCAGAGCCAAGATTGCGAATTTCGGTGTTGCGAGGATTCTTGATGAAGGTGATCTTGATCTTCAGTTAACAAGACATGTTGAAGGAACACAAGGCTACTTAGCTCCAGAGTATGTGGAGAATGGAGTCATTACTTCGAAACTAGACGTGTTTGCTTTTGGAGTTGCGGTTCTTGAGCTTCTTTCGGGGAGAGAAGCAGTAACGATACATAAGAAGAAGGAAGGAGAAGAAGAAGTGGAGATGTTGTGTAAAGTGATAAACAGTGTGCTTGGAGGAGAGAATGTGAGAGAGAAGTTAAAAGAGTTTATGGATCCATCTCTAGGGAATGAGTATCCGTTGGAGCTGGCTTACACCATGGCTCAGCTTGCTAAGAGCTGTGTCGCAACTGATCTTAACTCGCGTCCATCTGTCACTCAGGTTCTAACCACGCTCTCAATGATCGTCTCCTCCTCCATCGATTGGGAGCCTTCTGATGACCTTCTTCGTTCCGGCTCTCTTGGCAACGACTACAAAGACGATGACGACAAAGACTACAAAGACGATGACGACAAAGACTACAAAGACGATGACGACAAAGCGTCCAATAACGGGAACGGCAGGAACGGGATGGTTTCAAAAGGTGAGGAACTTTTCACCGGCGTGGTACCGATTTTGGTCGAATTAGATGGCGACGTAAACGGTCACAAATTCAGTGTCTCCGGGGAGGGTGAGGGTGATGCAACCTACGGCAAGCTTACGTTAAAATTCATCTGCACAACCGGTAAGCTACCGGTCCCTTGGCCTACACTGGTGACAACACTGACATACGGGGTACAGTGCTTTAGTCGTTATCCAGATCATATGAAACAACATGATTTTTTTAAGAGTGCGATGCCAGAAGGGTATGTTCAAGAACGAACGATATTTTTCAAGGACGATGGGAATTACAAGACTAGAGCAGAGGTAAAGTTTGAGGGTGATACTCTCGTCAACCGAATCATCTTGAAGGGTTGCTTTGCAAAAGGTACCAATGTTTTGATGGCCGATGGGTCTATCGAATGTATTGAGAACATAGAAGTAGGGAACAAGGTCATGGGAAAGGACGGTCGACCGCGAGAAGTAATAAAATTGCCTAGAGGGAGGGAGACTATGTACTCAGTGGTGCAAAAAAGTCAACATAGGGCCCACAAATCTGATAGCAGCAGGGAAGTTCCTGAATTATTAAAGTTCACATGTAACGCCACCCATGAGCTAGTAGTCCGTACGCCGCGATCAGTGCGTCGACTTAGCCGAACTATTAAGGGCGTTGAATACTTCGAGGTCATAACTTTTGAAATGGGTCAGAAAAAAGCTCCTGATGGGAGAATTGTCGAGCTGGTAAAGGAGGTCAGTAAATCATATCCGATAAGTGAAGGCCCCGAACGAGCGAACGAACTAGTTGAGAGCTATCGTAAAGCGTCTAACAAAGCCTACTTTGAATGGACTATAGAAGCCCGTGACCTATCCCTGTTGGGTTCTCATGTCCGTAAGGCAACATACCAGACGTACGCCCCTATACTGTATTGAATATGAAGATGAAGATGAAATATTTGGTGTGTCAAATAAAAAGCTTGTGTGCTTAAGTTTGTGTTTTTTTCTTGGCTTGTTGTGTTATGAATTTGTGGCTTTTTCTAATATTAAATGAATGTAAGATCTCATTATAATGAATAAACAAATGTTTCTATAATCCATTGTGAATGTTTTGTTGGATCTCTTCTGCAGCATATAACTACTGTATGTGCTATGGTATGGACTATGGAATATGATTAAAGATAAGATGGGCTCATAGAGTAAAACGAGGCGAGGGACCTATAAACCTCCCTTCATCATGCTATTTCATGATCTATTTTATAAAATAAAGATGTAGAAAAAAGTAAGCGTAATAACCGCAAAACAAATGATTTAAAACATGGCACATAATGAGGAGATTAAGTTCGGTTTACGTTTATTTTAGTACTAATTGTAACGTGAGACTACGTATCGGGAATCGCCTAATTAAAGCATTAATGCGAACCTGATTAGATTCACCGACCCTCCTATCGTGTCGACCTTTCTGTTTCTTAGAATTTTTTGGTAGTCTATGTACTAATAATGTCAGCTTCGTATTTATTTCATAAGCAATTTGCATTTGCAATTTGTTTTTTACTTTTATTTTTATTGTATTGTGGAATGTGGACTCGTACCAACATGAAGTTATATACCACCAAAAAAATTACAGTTAGTCAAAAGATTCACGAGTGAGAGCTACTTATGATTGTCTTTTACGTATATGTCTAATTGTCTATTTGCTCAATAATCTTTGTACTTTCTTTTGTCGTTGATAAAATCACAAAGTTCCAAAAGTAATCGAATGATTTGCTTTTAAGAAAAGAAGAGCTCAATAATTCAACATATATCTGTACACAGACGGACGTGGGCAGGTG

**Supplemental Figures**


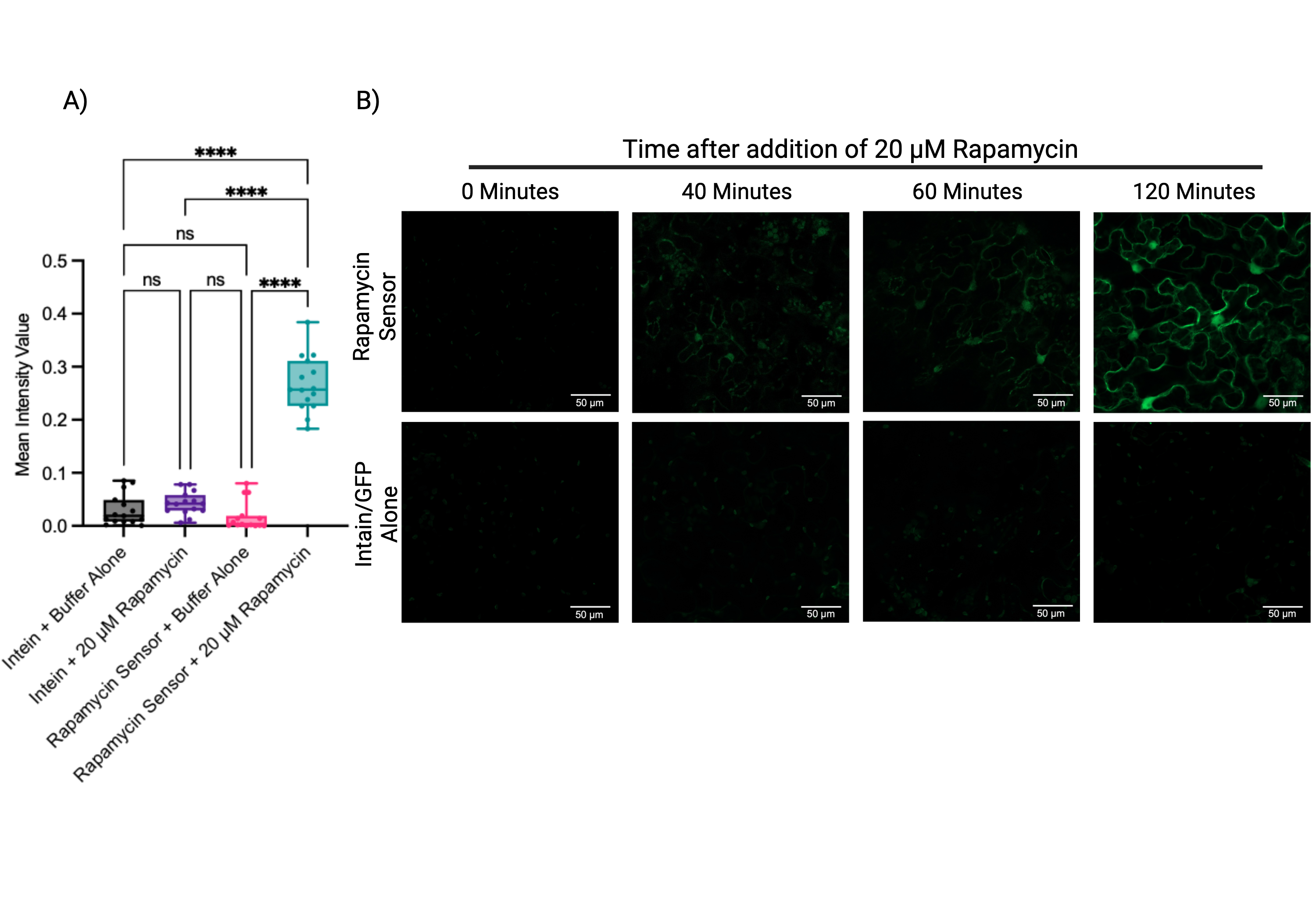


Figure S1. GFP intensity quantification and rapamycin time course experiment.

**(A)** Box plots comparing GFP intensity of 15 leaf samples across n = 3 biological replicates (5 leaves per replicate) of the rapamycin sensor experiment performed for Figure 1B. Statistically compared using Kruskal-Wallis one-way ANOVA with Dunn’s multiple comparison test post hoc analysis. **** = P< 0.0001, ns = P > 0.05. **(B)** Time course experiment demonstrating that GFP signal can be detected in as little as 40 minutes in tobacco leaves after addition of rapamycin to leaves expressing the rapamycin sensor or intein-onlu control. Scale bars are 50μm.


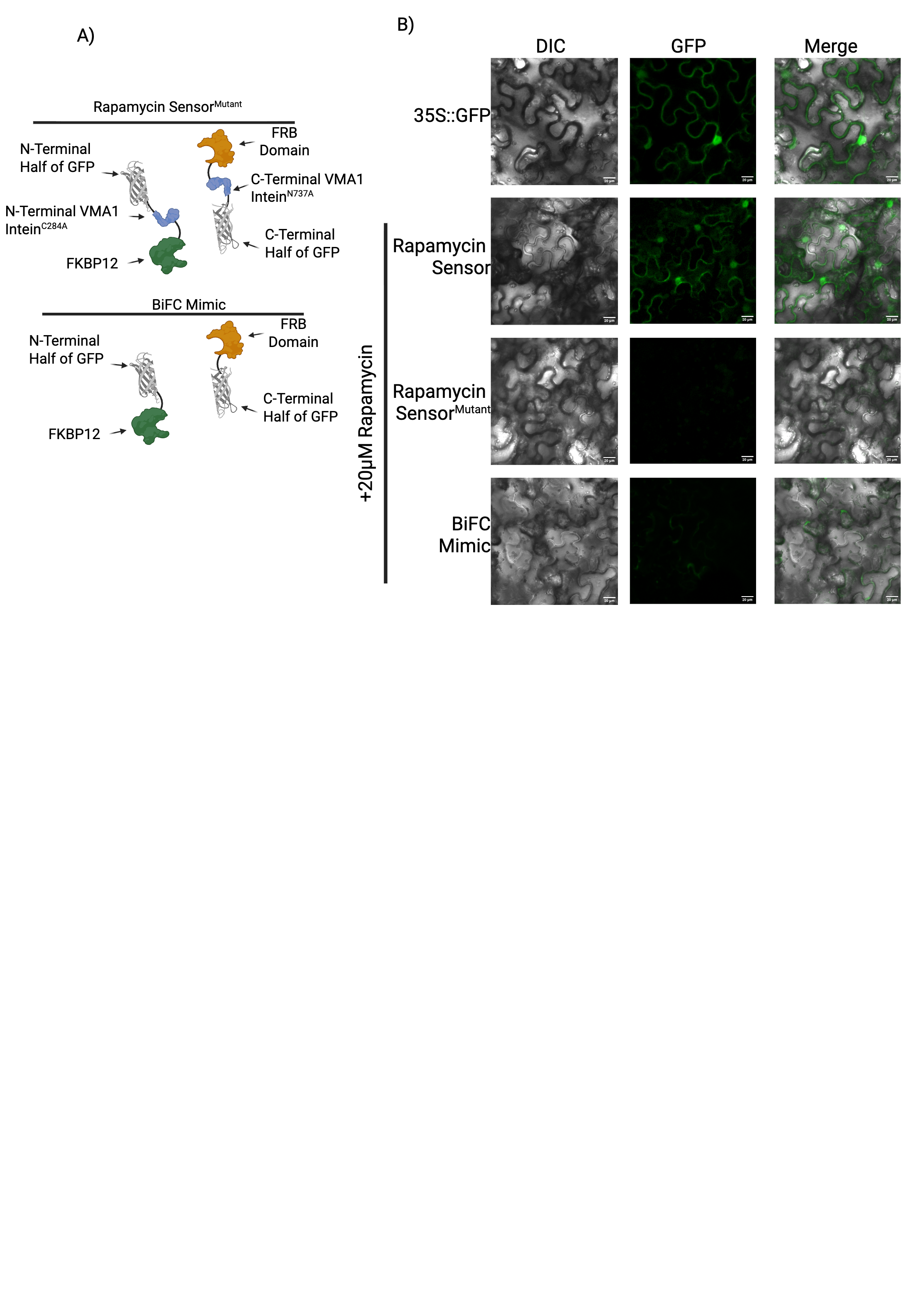


Figure S2. Mutant rapamycin sensor and BiFC control compared to rapamycin sensor.

**(A)** Model depicting the mutant rapamycin sensor and BiFC mimic constructs being tested. Image made using BioRender.com **(B)** GFP signal of tobacco leaves expressing either GFP positive control, rapamycin sensor, mutant rapamycin sensor, or BifC mimic with 20 μM rapamycin. Scale bars are 20μm.


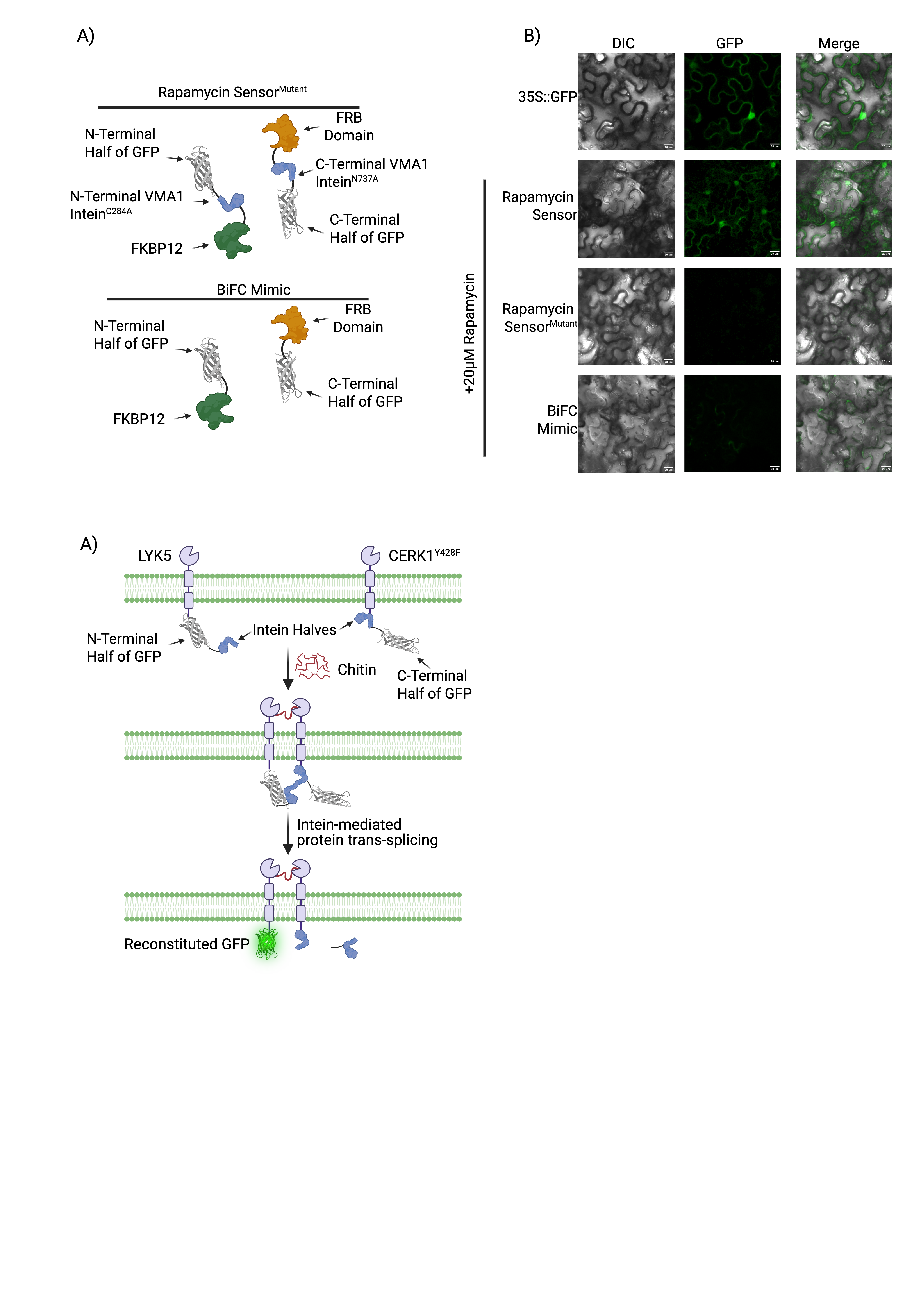


Figure S3. Depiction of chitin sensor based production of eGFP.

**(A)** Model depicting the chitin sensor binding chitin through LYK5 and CERK1^Y428F^ heterodimerization and forming eGFP through intein-mediated trans-splicing. Image made using BioRender.com.


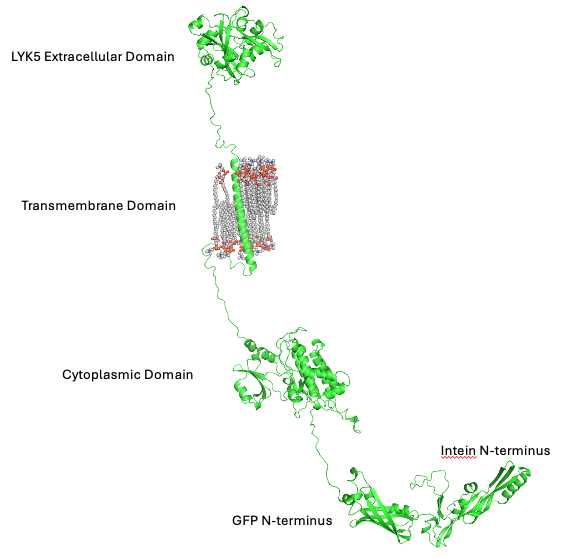


Figure S4. AlphaFold3 model of LYK5 – FLAG tag – N-terminal intein – N-terminal GFP.

The membrane environment was modeled with a small number of 1-palmitoyl-2-oleoyl-sn-glycero-3-phosphocholine (POPC) lipids (shown in grey sticks). Portions of the protein were manually moved to improve clarity (See Methods for details).


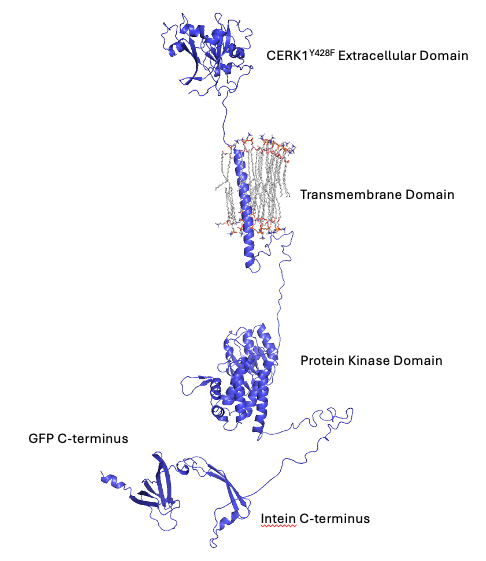


Figure S5. AlphaFold3 model of CERK1^Y428F^ – FLAG tag – C-terminal intein – C-terminal GFP.

The membrane environment was modeled with a small number of 1-palmitoyl-2-oleoyl-sn-glycero-3-phosphocholine (POPC) lipids (shown in grey sticks). Portions of the protein were manually moved to improve clarity (See Methods for details).


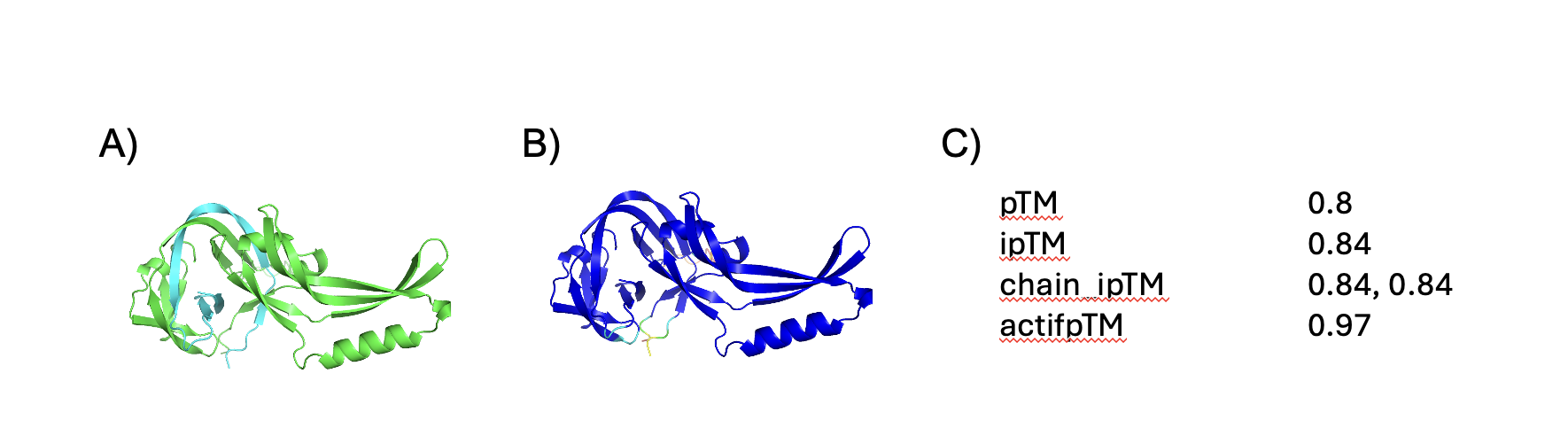


Figure S6. AlphaFold3 model of the split intein complex.

(**A**) Split intein model colored by chain. (**B**) Split intein model colored by per-atom predicted local distance difference test (pLDDT); Dark blue: pLDDT > 90; cyan: 80-90; green: 70-80; yellow: 60-70; orange: 50-60; red: pLDDT < 50. (**C**) Selected confidence metrics for the model.


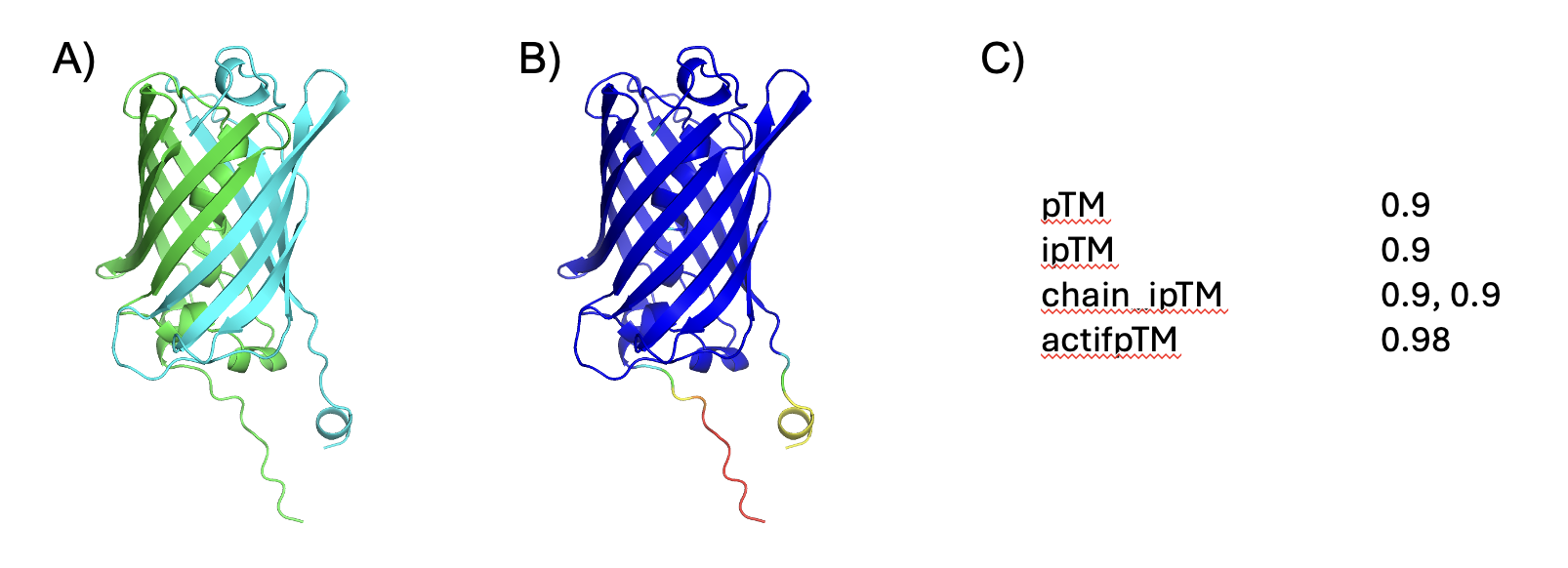


Figure S7. AlphaFold3 model of the split GFP complex.

(**A**) model colored by chain (green: N-terminal fragment, cyan: C-terminal fragment). (**B**) model colored by per-atom predicted local distance difference test (pLDDT); Dark blue: pLDDT > 90; cyan: 80-90; green: 70-80; yellow: 60-70; orange: 50-60; red: pLDDT < 50. **(C)** Selected confidence metrics for the model.


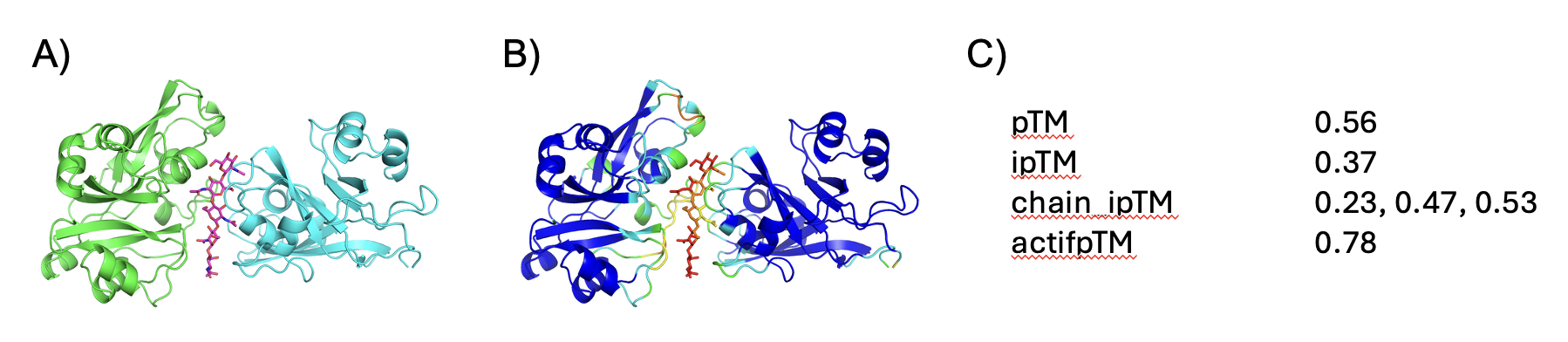


Figure S8. AlphaFold3 model of the extracellular domains of LYK5 and CERK1^Y428F^ with a bound chitin pentamer.

(**A**) Model colored by chain (green: LYK5, cyan: CERK1; magenta: chitin). **(B)** Model colored by per-atom predicted local distance difference test (pLDDT); Dark blue: pLDDT > 90; cyan: 80-90; green: 70-80; yellow: 60-70; orange: 50-60; red: pLDDT < 50. **(C)** Selected confidence metrics for the model. Note that model confidence is not expected to correlate with binding affinity but is instead likely indicative of limitations in AlphaFold3 training data.


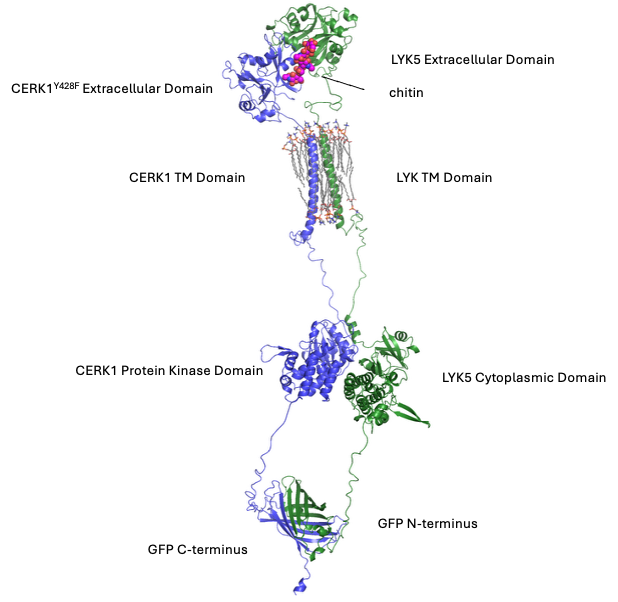


Figure S9. AlphaFold3 model of a chitin sensor lacking a split intein.

A bound chitin pentamer is shown in magenta spheres. The membrane environment was modeled with a small number of 1-palmitoyl-2-oleoyl-sn-glycero-3-phosphocholine (POPC) lipids (shown in grey sticks). Portions of the protein were manually moved to improve clarity (See Methods for details). TM = transmembrane domain.


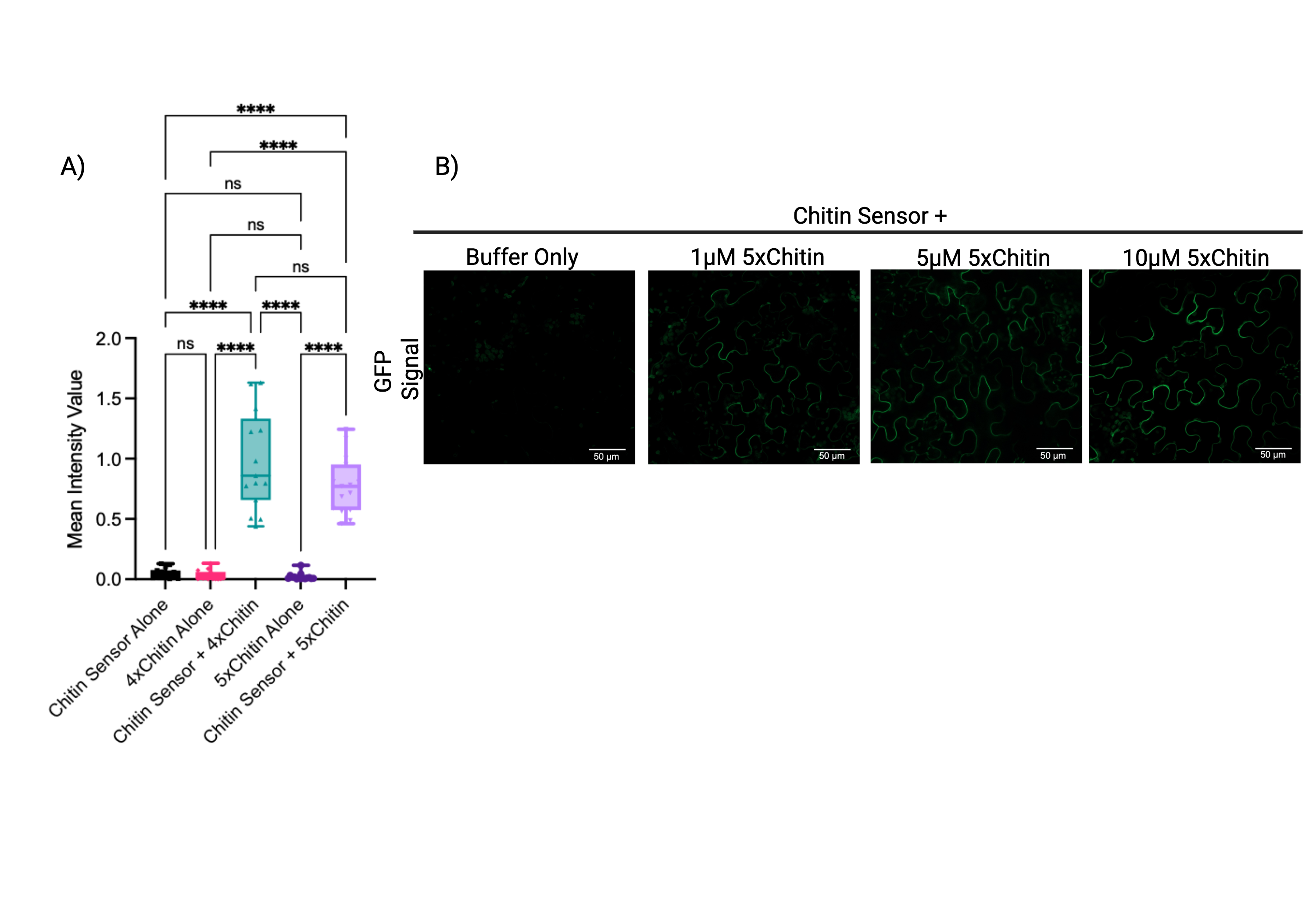


Figure S10. Chitin dilution experiment along with GFP intensity comparison.

**(A)** GFP intensity values across 15 leaf samples from n = 3 biological replicates (5 leaves per replicate) from the chitin sensor experiment shown in Figure 1E. Statistically compared using Kruskal-Wallis one-way ANOVA with Dunn’s multiple comparison test post hoc analysis. **** = P < 0.0001, ns = P > 0.05. **(B)** Leaf images of the chitin sensor with various concentrations of 5xchitin polymer. Scale bars are 50μm.


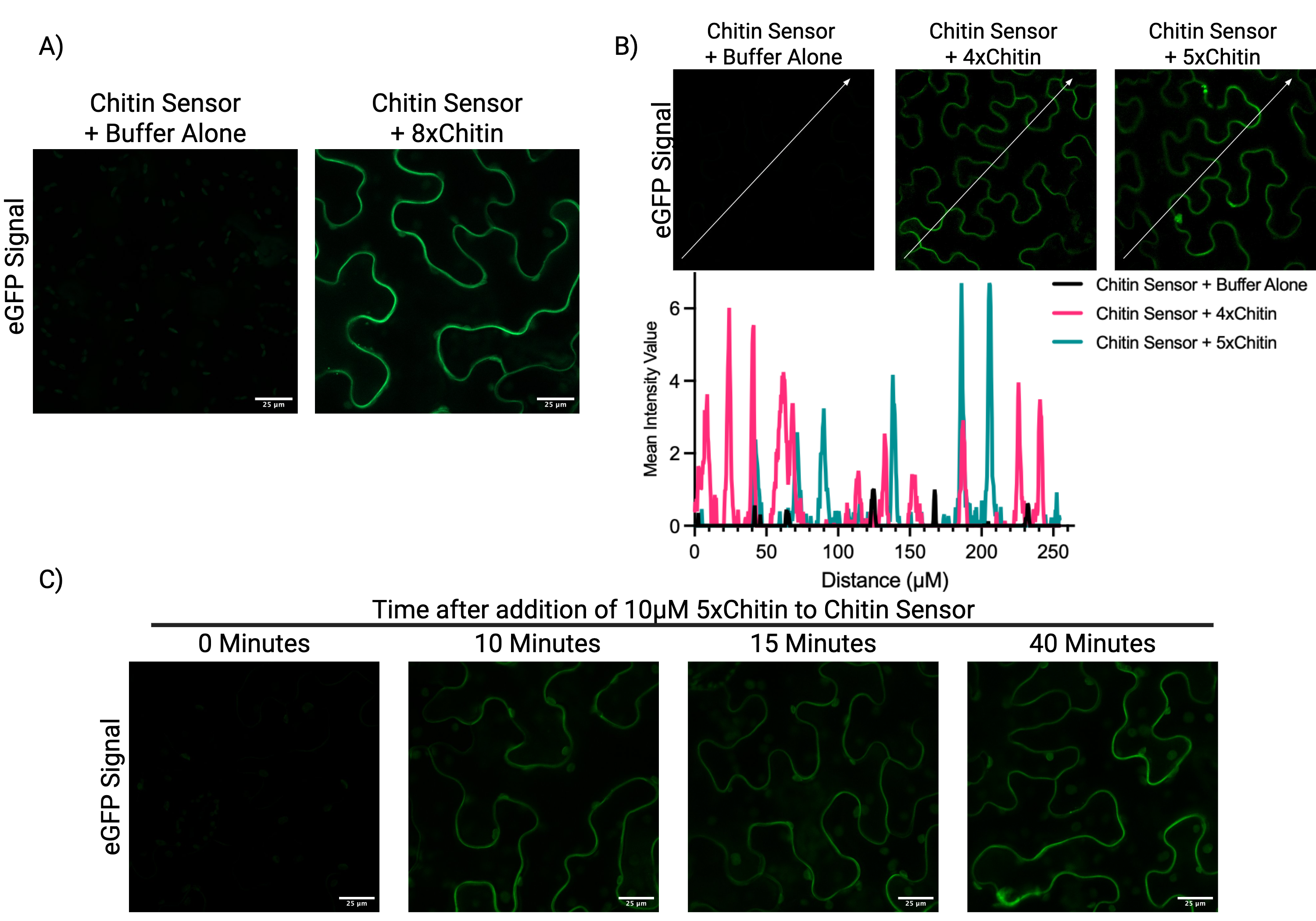


Figure S11. Chitin sensor time course experiment, 8xChitin polymer experiment, and intensity comparison.

**(A)** Representative images of the chitin sensor with buffer alone or 10 µM 8xchitin polymer. Scale bars are 25μm. **(B)** Comparison of eGFP intensities for the Chitin sensor with buffer alone, 4xchitin polymer, and 5xchitin polymer. eGFP intensity plotted across the distance of the white line for each condition demonstrates the difference in eGFP intensities when the chitin sensor detects chitin. Color of lines is as follows: black is chitin sensor plus buffer alone, red is chitin sensor plus 4xchitin, and green is chitin sensor plus 5xchitin. **(C)** Time course experiment for chitin sensor eGFP signal in tobacco leaves using 5xchitin polymer, demonstrating eGFP signal in as little as 10 minutes. Scale bars are 25μm.

**References**

Anraku, Y. and Satow, Y. (2009) *Reflections on protein splicing: structures, functions and mechanisms*. *Proc Jpn Acad Ser B Phys Biol Sci*, **85**, 409–421.

Čermák, T., Curtin, S.J., Gil-Humanes, J., Čegan, R., Kono, T.J.Y., Konečná, E., et al. (2017) *A Multipurpose Toolkit to Enable Advanced Genome Engineering in Plants*. *The Plant Cell*, **29**, 1196–1217.

Sparkes, I.A., Runions, J., Kearns, A., and Hawes, C. (2006) *Rapid, transient expression of fluorescent fusion proteins in tobacco plants and generation of stably transformed plants*. *Nat Protoc*, **1**, 2019–2025.

Varga, J.K., Ovchinnikov, S., and Schueler-Furman, O. (2025) *actifpTM: a refined confidence metric of AlphaFold2 predictions involving flexible regions*. *Bioinformatics*, **41**, btaf107.

Abramson, J., Adler, J., Dunger, J., Evans, R., Green, T., Pritzel, A., et al. (2024) *Accurate structure prediction of biomolecular interactions with AlphaFold 3*. *Nature*, **630**, 493–500.

Mirdita, M., Schütze, K., Moriwaki, Y., Heo, L., Ovchinnikov, S., and Steinegger, M. (2022). *ColabFold: making protein folding accessible to all*. *Nat Methods*, **19**, 679–682.

Steinegger, M. and Söding, J. (2017) *MMseqs2 enables sensitive protein sequence searching for the analysis of massive data sets*. *Nat Biotechnol*, **35**, 1026–1028.
